# Preventing Introduction of Livestock Associated MRSA in a Pig Population – Benefits, Costs, and Knowledge Gaps from the Swedish Perspective

**DOI:** 10.1371/journal.pone.0122875

**Published:** 2015-04-29

**Authors:** Sören Höjgård, Olov Aspevall, Björn Bengtsson, Sara Hæggman, Maria Lindberg, Kristina Mieziewska, Svante Nilsson, Helle Ericsson Unnerstad, Diana Viske, Helene Wahlström

**Affiliations:** 1 Swedish University for Agricultural Sciences, and AgriFood Economics Centre, Uppsala, Sweden; 2 Public Health Agency of Sweden, Solna, Sweden; 3 National Veterinary Institute, Uppsala, Sweden; 4 Federation of Swedish Farmers, Stockholm, Sweden; 5 Swedish Animal Health Service, Uppsala, Sweden; 6 Swedish Board of Agriculture, Jönköping, Sweden; Rockefeller University, UNITED STATES

## Abstract

Antibiotic resistance is a growing concern in human, as well as in veterinary medicine. Part of the problem concerns how to respond to the risk presented by animal reservoirs of resistant bacteria with the potential of spreading to humans. One example is livestock associated methicillin-resistant *Staphylococcus aureus* (LA-MRSA). In countries where LA-MRSA is endemic in the pig population, people in contact with pigs have a higher risk of being colonised with LA-MRSA, and persons from this group are subjected to precautionary measures when visiting health care facilities. In the present study, it is assumed that, if LA-MRSA was introduced to the Swedish pig population, the prevalence in the risk groups would be the same as in Denmark or the Netherlands (two countries with low human prevalence that have implemented measures to detect, trace and isolate human LA-MRSA cases and, therefore, have comprehensive data with good coverage regarding prevalence of LA-MRSA), and that similar interventions would be taken in Swedish health care facilities. It is also assumed that the Swedish pig population is free of MRSA or that the prevalence is very low. We analyse if it would be efficient for Sweden to prevent its introduction by testing imported live breeding pigs. Given that quarantining and testing at import will prevent introduction to the pig population, the study shows that the preventive measures may indeed generate a societal net benefit. Benefits are estimated to be between € 870 720 and € 1 233 511, and costs to € 211 129. Still, due to gaps in knowledge, the results should be confirmed when more information become available.

## Introduction

Methicillin-resistant *Staphylococcus aureus* (MRSA) is resistant to most betalactam antibiotics, which includes the antibiotics of choice for treatment of staphylococcal infections. MRSA may colonise animals or humans without the carrier becoming ill but can also cause severe infections that are difficult to treat. Since its discovery in 1961, MRSA has become a major infection control problem in hospitals worldwide [1].

In the last decade, livestock-associated MRSA (LA-MRSA) of clonal complex (CC) 398, has become increasingly more common among pigs in several countries [2]. In most cases, pigs are only carriers without symptoms but constitute a reservoir with risk of spread to humans. In Sweden, however, LA-MRSA is believed to be rare in pigs. In the latest survey, including all nucleus and multiplying herds, MRSA was not found [3]. Moreover, in three earlier Swedish surveys, in 2007, 2008 and 2010, MRSA was detected only once, in a sample from pigs at slaughter [4]. The favorable situation is probably the result of several factors, but the limited import of live pigs is likely the most important.

Animal carriage of MRSA is a problem in all countries where colonised livestock is present, and may be of particular significance in countries with a low prevalence of MRSA in humans [5]. Examples are Denmark, the Netherlands, and Sweden where the total incidence per 100 000 inhabitants in 2013 were 37.4, 23.1, and 25.4, respectively [6–8]. In Denmark the human incidence of LA-MRSA has increased substantially from 0.8 per 100 000 (42 cases in the population) in 2009 to 11.6 per 100 000 (634 cases in the population) in 2013 [6]. In the Netherlands, human incidence was 7.6 per 100 000 in 2011 [9]. In Sweden the incidence of LA-MRSA in humans has so far been very low, 0.09 per 100 000 in 2013 [8]

In countries where LA-MRSA is widespread among pigs, human carriage is much more common in persons in contact with live pigs, and their household members, than among people in the community [10–12]. Some studies suggest that the nosocomial transmission rate of LA-MRSA is lower than for other types of MRSA [13, 14]. Also, person to person spread in the community appears to be rare although it cannot be ruled out as recently discussed [15]. This suggests that LA-MRSA in pigs primarily constitutes a risk to persons in close contact with the animals. However, in these studies some important factors related to transmission, e.g. rate of carriers vs clinical infections in the different groups, rate of risk factors for transmission in the different groups, and the distribution of the groups regarding type of hospital are lacking. Furthermore, in 2012, 21 percent of the MRSA CC398 cases in Denmark were persons without known direct or household contacts with live pigs [16]. Based on this, the results must be interpreted with caution.

The societal costs of LA-MRSA include costs in human health care related to LA-MRSA. In Sweden, findings of MRSA in humans and animals are notifiable [17, 18]. In humans, the physician is obliged to trace the source of infection and advise the patient on how to avoid further spread. Persons who know that they carry MRSA shall declare this when visiting a health care facility. Such patients are managed according to special recommendations regarding diagnostic tests, precautionary measures, and non-standard antibiotic treatment. In addition, there may be costs outside the health care sector such as production losses caused by infections, welfare losses caused by restrictions on the activities of carriers, pain and anxiety among carriers, and possibly loss of lives.

For a country free of LA-MRSA in the pig population, implementing measures to prevent its introduction may, therefore, increase societal welfare. The condition is that the reduction in the LA-MRSA related costs is larger than the costs of the preventive mea-sures themselves [19, 20]. To our knowledge, only one study has been done, in Norway [21], but no published scientific report is available. Hence, the purpose of the present paper is to investigate if available data allow an answer under Swedish conditions

Because LA-MRSA is widespread in pig production, including breeding herds, abroad [22], the Swedish Farmer’s Disease Control Programme (SDS), after consulting the Swedish National Veterinary Institute (SVA), has issued recommendations aiming at preventing introduction of LA-MRSA to the top of the Swedish breeding pyramid. SDS is a veterinary body founded by the industry to provide advice on biosecurity measures and recommend additional testing when importing farm animals, semen and embryos, to Sweden. The advice from SVA implies that imported breeding boars are quarantined and tested for MRSA by selective bacteriological culture. Each animal is to be sampled three times by skin swabs and in addition, environmental samples are taken twice from the quarantine. It is also advised to test each batch of imported fresh semen. If MRSA is found, it is advised not to introduce live animals or semen in the country. Import to Sweden of other pigs has been very limited in the last five years and restricted to about 6000 weaner piglets from Finland (personal communication SDS). In Finland LA-MRSA has been found in pigs but the occurrence in farrowing farms is reported to be limited [23]. Moreover, the Swedish farms importing the pigs have been finisher farms only and the farmers have been given advice on biosecurity measures to avoid introduction of LA-MRSA. This implies strict age-sectioning (all in—all out system), which means that all weaning/fattening pigs in a given farm are introduced at the same time and stables are disinfected before the next batch is introduced (personal communication SDS). Contrary to breeding herds, which sell live animals to other herds, fattening herds only send animals to slaughter. Thus, it is not likely that LA-MRSA would contaminate the general Swedish pig population if introduced through these imports of weaning/fattening pigs.

If these preventive measures are successful, fewer persons would be at risk of becoming colonised than if LA-MRSA was introduced among pigs in Sweden. Accordingly, the costs related to MRSA would also be lower. On the other hand, following the SVA-advices imply costs for quarantining and testing imported pigs/semen, destruction of colonised pigs/semen, and lower revenues in pig production due to reduced supply of genetic material caused by the destruction of pigs/semen.

## Materials and Methods

In this study it is assumed that the preventive measures are 100 percent effective, that is, they completely prevent the introduction of LA-MRSA among Swedish pigs. It is acknowledged that this may be questioned and we return to the issue in the discussion.

Without prevention, the prevalence of LA-MRSA in Swedish pigs is assumed to increase over time and eventually reach some steady state level, approximated by the current prevalence in the Netherlands and Denmark. Human prevalence in the risk group is likely to be proportional to that in pigs. Hence, it would seem natural to investigate if the sum of discounted annual benefits of the measures suggested by SVA, eventually, would cover the sum of their discounted annual costs and how long it would take for them to do so [19, 20]. However, this requires information on how LA-MRSA is spread in the pig population and how long it would take to reach steady state. As this is not available, we analyse if the annual benefits of the suggested measures could be expected to cover their annual costs when steady state *has* been reached. This implies that there is no need to discount costs and benefits as they occur in the same year.

*The societal benefits* of the measures are the avoided costs in the Swedish risk group. In order not to overestimate these costs, the risk group is assumed to consist of persons in close contacts with pigs only—pig farmers and their employees, slaughterhouse workers, pig transporters, veterinarians, and their family members—as in the Dutch guidelines [24]. The prevalence of LA-MRSA in the Dutch and Danish risk groups are used to approximate the expected human prevalence in the Swedish risk group in steady state. The analysis is limited to costs in the health care sector as there is not enough information to estimate the frequency of other events, i.e. restrictions on carrier activities and deaths from LA-MRSA. Furthermore, it is assumed that the incidence of infections caused by *Staphylococcus aureus* (*S*. *aureus*) in the Swedish risk group would remain the same if LA-MRSA were introduced. The societal costs of LA-MRSA are, therefore, the excess costs that would occur for patients infected by LA-MRSA compared to patients infected by antibiotic susceptible *S*.*aureus*.

*The societal costs* of the preventive measures suggested by SVA are analysed assuming imports of live boars from Norway. It is acknowledged that this is a special case and, if the probability of testing positive for LA-MRSA is higher than that for Norwegian boars, the costs of the measures will be underestimated.

### Potential cost savings in human health care

**Fig 1:**
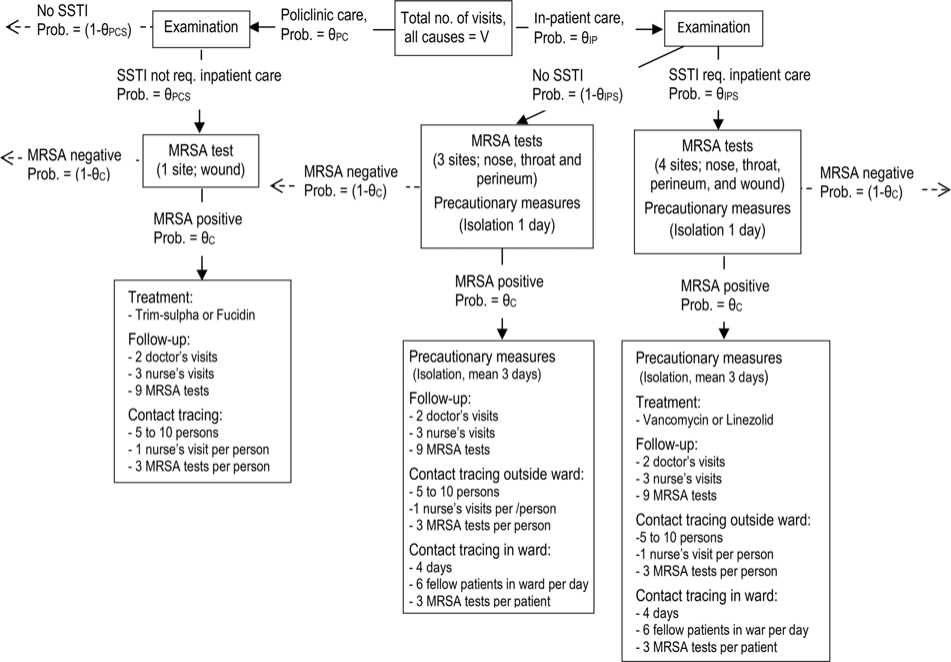
Event-tree for the risk group with respect to LA-MRSA interventions.

Danish guidelines define persons in contact with pigs as a risk group for MRSA in the Danish health care and specify how to treat persons from the risk group seeking health care [25]. We assume that a risk group would be defined in the same way by the Swedish health care if LA-MRSA should become endemic in the pig population. The events expected to cause costs related to LA-MRSA in human health care are illustrated in Fig 1 below.

When a person in the risk group visits a policlinic (which serve as “gate keepers” in Swedish health care), it is first decided whether she can be treated policlinically (probability *θ*_*PC*_) or requires inpatient care (probability *θ*_*IP*_). Note that *θ*_*IP*_ *=* (1-*θ*_*PC*_), however, they are given separate labels to facilitate notation.

Given *policlinic treatment*, only patients with skin or soft tissue infections (SSTI) are of interest (probability *θ*_*PCS*_). These patients will be tested in one site (wound) [26, 27] and, if LA-MRSA-positive (probability *θ*_*C*_), treated with trimethoprim-sulphonamide or fusidic acid [27, 28], and subject to follow-up measures (two doctor’s visits, three nurse’s visits and nine MRSA-tests per patient), and to contact tracing (three MRSA-tests and one nurse’s visit each for five to ten persons that have been in close contact with the patient; personal communication, Stockholm County Council (SLL)). Contact tracing may reveal new carriers. However, as data to estimate this probability is lacking, costs generated by them are not included.

Given *inpatient care*, patients referred *for SSTI*, implying that they have *severe* SSTI, (probability *θ*_*IPS*_) are tested at four sites (nose, throat, perineum and wound) and, awaiting the results, which usually takes one day, isolated with extra hygienic precautions (i.e. specially dedicated personnel) [25, 26]. MRSA-positive patients (probability *θ*_*C*_) remain in isolation for the rest of their stay—on average three days [29], and are treated with infusions of vancomycin or linezolid [27]. The same measures for follow-up and contact tracing outside ward as in policlinic care will be undertaken (personal communication SLL). Contact tracing inside ward will also be performed—subjecting patients discharged from the same ward (on average six per day) to three MRSA tests each, every day the MRSA-positive patient remains at the ward (i.e. a total of 6×4×3 = 72 tests; personal communication, SLL). Contact tracing may reveal new cases but, on the same grounds as before, costs generated by them are not included.

Patients referred to *inpatient care for other causes* (probability (1-*θ*_*IPS*_)) are tested at three sites (nose, throat, and perineum) and isolated awaiting the results. Positive results (probability *θ*_*C*_) are assumed to imply that the person is a carrier only (i.e. no infection). Hence, they are not treated with antibiotics but remain in isolation for the rest of their hospital stay, and subjected to follow-up measures (same as in policlinic care). The same measures for contact tracing both inside and outside ward as for inpatients with SSTI caused by MRSA are also applied (personal communication, SLL; [29]. Again, contact tracing may reveal new cases but they are not included. Furthermore, costs caused by nosocomial transmission in inpatient care are not accounted for.

### Size of risk group, annual number of visits, and probabilities

The size of the risk group (*N*) is estimated based on information on the number of pig farmers and their employees, slaughterhouse workers, and transporters in contact with live pigs obtained from the Swedish Animal Health Service (SvDHV). Data on the number of veterinarians in contact with live pigs are from SvDHV, the Swedish National Food Agency (SLV), and the Swedish Board of Agriculture (SJV). The number of persons sharing household with someone from any of the sub groups is estimated using data on the number of persons in the average Swedish household from Statistics Sweden. This results in a risk group consisting of about 6 000 persons (Table 1).

**Table 1:** The Swedish risk group.

Variable	Description	Value
*N*_*1*_	Pig-farmers and employees of pig farms^a^	2 500
*N*_*2*_	Slaughterhouse workers in contact with live pigs^a^	100
*N*_*3*_	Pig transporters^a^	200
*N*_*4*_	Veterinarians in contact with live pigs^a^ ^,^ ^b^ ^,^ ^c^	240
*N*_*5*_	Persons sharing household with someone in sub-groups *N*_*1*_ to *N*_*4*_ (i.e.$\sum_{i=1}^{4}N_{i}$)^d^	3 040
*N*	Total number of persons in the risk-group (i.e.$\sum_{i=1}^{5}N_{i}$)	6 080

^a)^SvDHV.

^b)^Swedish National Food Agency.

^c)^SJV.

^d)^ According to Statistics Sweden, the average Swedish household consists of two persons.

The health related behavior of the risk group is assumed to be similar to that of the general Swedish population. Accordingly, information on the number of policlinic visits per 100 000 persons (0–64 years old) for the period 2001–2011 (obtained from SKL and the Swedish Board of Health and Welfare, see Table A in S1 Information), is used to estimate the *expected annual number of visits* in the risk group (*V*), and described by a Pert distribution [30], using the lowest observed value as the minimum, the median as the most likely, and the highest observed value as the maximum (see Table 2).

**Table 2:** Expected annual number of visits and probabilities in Fig 1.

Variable	Description	Estimation/Value
*V*	Expected annual number of policlinic visits (all causes)^a^	pert (8 578, 9 307, 10 142)
*θ*_*PC*_	Probability of a policlinic treatment, given visit, all causes.^a^	pert (0.9292, 0.9336, 0.9381)
*θ*_*PCS*_	Probability of SSTI, given policlinic treatment.^b^	pert (0.0133, 0.0152, 0.0165)
*θ*_*IP*_	Probability of in-patient care, given visit, all causes.^a^	pert (0.0618, 0.0664, 0.0708)
*θ*_*IPS*_	Probability of SSTI, given in-patient care.^a^	pert (0.0042, 0.0044, 0.0049)
*θ*_*CNL*1_	Prevalence of LA-MRSA in pig-farmers and their employees in the Netherlands (three studies).^c^	discrete [{beta (7, 21), beta (29, 71),beta (14, 37)}; {(0.1, 0.57, 0.28)}]
*θ*_*CNL*5_	Prevalence of LA-MRSA in pig-farmers’ families in the Netherlands (one study).^d^	beta (6, 30)
*θ*_*CNL*2,3_	Prevalence of LA-MRSA in slaughterhouse workers and pig transporters in the Netherlands (three studies).^e^	discrete [{beta (15, 80), beta (5, 33), beta (4, 32)}; {(0.57, 0.22, 0.21)]
*θ*_*CNL*4_	Prevalence of LA-MRSA in veterinarians in the Netherlands (one study).^f^	beta (7, 21)
	Proportion of Swedish risk group belonging to, respectively, sub-groups *N*_*1*_, *N*_*2*_, *N*_*3*_, *N*_*4*_, and *N*_*5*_.^g^	Π_s1_ = 0.412: Π_s2_ *=* 0.0165: Π_s3_ = 0.0329: Π_s4_ = 0.0395: Π_s5_ = 0.5
*θ*_*CNL*_	Probability of LA-MRSA in the Swedish risk-group based on prevalence in the Netherlands.	discrete {[*θ*_*CNL*1_, *θ*_*CNL*2, 3_, *θ*_*CNL*4_, *θ*_*CNL*5_ [Π_*s*1_, (Π_*s*2_+Π_*s*3_), Π_*s*4_, Π_*s*5_]}
*N*_*DK*_	Number of persons in the Danish risk-group.^h^	22 740
*N*_*RDK*_	Number of reported LA-MRSA cases in the Danish risk-group.^h^	149
	Expected annual number of MRSA-tested visits in the Swedish risk-group	749
	Expected annual number of visits per person in the Swedish risk-group, given at least one visit.	2.19
	Expected annual number of persons in the Swedish risk-group visiting a health care facility and being tested for MRSA.	342
*λ*	Expected share of risk-group visiting a health care facility and being tested for MRSA.	0.056
*θ*_*CDK*_	Probability of LA-MRSA in Swedish risk-group based on no. of cases detected in Denmark and size of Danish risk-group.	0.08

^a)^Estimated using data from Swedish Board of Health and Welfare and SKL.

^b)^Estimated using data from Andre et al [31] and Swedish Board of Health and Welfare.

^c)^ Voss et al. [32], Wulf et al. [37], van den Broek et al. [33], van Cleef et al [34].

^d)^van den Broek et al. [33].

^e)^van Cleef et al. [35], and Gilbert et al [36].

^f)^Wulf et al [37].

^g)^Computed from Table 1 above.

^h)^Personal communication, FOI.

The *probability of policlinic treatment* (all causes = *θ*_*PC*_) is estimated as the annual proportion of all visits that are primary care visits, using observations for the period 2001–2011 (obtained from SKL, see Table A in S1 Information). As for expected number of visits (*V*), the probability is described by a Pert distribution. As referral to inpatient care (*θ*_*IP*_) is the complement to policlinic treatment, the *probability of referral to inpatient care* is simply (1-*θ*_*PC*_).

The *probability of SSTI*, given policlinic care, (*θ*_*PCS*_) is estimated similarly (i.e. as the share of primary care that has SSTI, and also described by a Pert distribution), but using information for the years 2000, 2002, and 2005 from André et al. [31] (see Table A in S1 Information).

The *probability of SSTI*, given inpatient care, (*θ*_*IPS*_) is estimated as the share of SSTIs in all inpatient care episodes using observations for the period 2001–2011 (Swedish Board of Health and Welfare, see Table A in S1 Information). As before, assumed to be described by a Pert distribution.

Two estimates of the *expected prevalence of MRSA* in the Swedish risk group (*θ*_*C*_) are used. First, it is assumed that it would be similar to the prevalence in the Dutch risk group (*θ*_*CNL*_), estimated using information on prevalence among Dutch pig farmers and their employees [32–34], family members of Dutch pig farmers and their employees [31], Dutch slaughterhouse workers and pig transporters [12, 35, 36], and among Dutch veterinarians [37] (see Table B in S1 Information). The prevalence in each study is described by a Beta-distribution (*n+1*, *n-s+*1), where *n* is the number of observations and *s* the number of positives. For sub groups with data from more than one study, prevalence is described by a discrete distribution—RiskDiscrete [30] {(*θ*_*CNL*1_, *θ*_*CNL*2_,…… *θ*_*CNLn*_), (Π_*NL*1_, Π_*NL*2_,…… Π_*NLn*_)} where the *θNLi*’s are the prevalence and the Π_*NLi*_’s the proportion of observations, in each study. Finally, the overall prevalence in the Swedish risk group is described by a RiskDiscrete distribution {(*θ*_*s*1,_ *θ*_*s*2,_ …… *θ*_*sn*_), (Π_*s*1,_ Π_*s*2,_ ……Π_*sn*_)} where the *θ*_*sj*_’s are the prevalence in each Dutch sub group and the Π_*sj*_’s the shares of the risk group belonging to each Swedish sub group (Table 2).

Second, expected prevalence is assumed to be similar to the prevalence in the Danish risk group (*θ*_*CDK*_). However, data for Denmark are scarce and only contain information on the annual number of *reported* human cases for a few years (personal communication, Department of Food and Resource Economics (FOI), Denmark). Hence, *θ*_*CDK*_, is estimated as:

$$\theta_{CDK}\;=\;\frac{N_{RDK}/\lambda }{N_{DK}}$$ (1)

where *N*_*DK*_ = no. of persons in the Danish risk group, *N*_*RDK*_ = no. of reported MRSA-positive persons in the Danish risk group, and *λ* = the proportion of the Danish risk group that is tested.

*λ is* assumed to be the same as in Sweden (for details, see S1 Information). This gives a *λ* of (342/6 080 = 0.056). Combining this with information on *N*_*RDK*_ and *N*_*DK*_, respectively, 149 and 22 740 persons in 2011 (personal communication FOI, Denmark), gives the estimate of *θ*_*CDK*_ reported in Table 2.

### Costs of MRSA-related health care interventions

Given the MRSA-related events in Fig 1, the excess costs of LA-MRSA could be summarised as follows:

1. costs for MRSA-tests per visit; *C*_*T*_ in policlinics, 3*C*_*T*_ in in patient care no SSTI, and 4*C*_*T*_ in in patient care given SSTI.
2. costs for interventions due to positive findings in *policlinic care*—i.e. excess costs for antibiotic treatment in policlinic care (*C*_*APC*_), costs for follow-up (*C*_*FU*_ = two doctors’ visits (2*C*_*DV*_) + three nurses’ visists (3*C*_*NV*_) + nine MRSA-tests (9*C*_*T*_)), and costs for contact tracing (*C*_*CT*_ = 7.5*C*_*NV*_×3*C*_*T*_ assuming that on average 7.5 persons will be tested).
3. costs for precautionary measures in *inpatient care* before MRSA status is known (*C*_*PM*_ = one day of isolation (*C*_*I*_) and more stringent hygienic measures (*C*_*H*_))
4. costs for interventions due to positive findings in *inpatient care*—i.e. excess costs for antibiotic treatment in inpatient care (*C*_*AIP*_), costs for precautionary measures (3*C*_*T*_), costs for follow-up (*C*_*FU*_), and costs for contact tracing outside (*C*_*CT*_) and inside (*C*_*CTI*_ = 4×6×3*C*_*T*_) ward

Costs in categories 1 and 3 are independent of prevalence as they concern interventions before the status of the patient is known. Information on costs used for the analyses are shown in Table 3.

**Table 3:** MRSA-related costs in Swedish health care (€, 2011 prices).

Variable	Description	Value
*C*_*T*_	Cost of diagnostic test for MRSA^a^	43.89
*C*_*DV*_	Cost of doctor’s visit for follow-up of MRSA in primary care^b^	463.73
*C*_*NV*_	Cost of nurse’s visit for follow-up of MRSA in primary care^b^	60.65
*C*_*FU*_	Cost of follow-up = (2*C*_*DV*_ + 3*C*_*NV*_ + 9*C*_*T*_)	1504.42
*C*_*CT*_	Cost of contact tracing outside inpatient ward = 7.5(*C*_*NV*_ + 3*C*_*T*_)	1923.20
*C*_*CTI*_	Cost of contact tracing inside inpatient ward = 4 × 6 × 3*C*_*T*_	3160.08
*C*_*I*_	Costs per day for isolation^c^	484.41
*C*_*H*_	Cost per day for stricter hygienic measures^c^	152.00
*C*_*APC*_	Average excess cost of antibiotic treatment for uncomplicated MRSA infections at policlinic facilities (trimetoprim-sulphonamide or fucidin acid instead of flucloxacillin)^d^	0
*C*_*AIP*_	Average excess cost of antibiotic treatment for severe MRSA infections in in-patient care (vancomycin or linezolid instead of cloaxcillin)^d^ = (0.8 × 0) + (0.2 × 373.50)	74.7
*T*_*IPS*_	Average length of hospital stay (days)^e^	4

^a)^County Council of Västra Götaland [38] and County Council of Örebro [39].

^b)^Västra Götalandsregionen [40].

^c)^Personal communication, County Council of Skåne.

^d)^Antibiotics recommended for policlinic treatment of MRSA-infections, trimethoprim-sulphonamide or fusidic acid [42], are no more costly than flucloxacillin, the antibiotic recommended for treatment of sensitive infections [28, 41]. As to antibiotics used for severe infections in inpatient care, vancomycin costs about the same as cloxacillin—recommended for treatment of severe sensitive infections [41]—linezolid, however, used in about 20 percent of MRSA cases (personal communication, Public Health Agency of Sweden), is € 374 more expensive than cloxacillin. Costs are based on prices from Swedish Pharmacies [41].

### Expected benefits—costs savings—of the preventive measures

The expected societal benefits of the preventive measures, *E*(*SB*_Prev_) are the costs that would have been incurred in the absence of the measures. That is, the costs caused by the events in Fig 1, multiplied by the probabilities of the respective events. Accordingly, the total expected benefits of the preventive measures can be expressed as:

$$E\left(SB_{\mathrm{Pr}ev}\right)=\left\{\begin{array}{l} V\times \left[\begin{array}{l} \left(\theta_{PC}\times \theta_{PCS}\times C_{T}\right)+\\ \left(\theta_{IP}\times \theta_{IPS}\times 4C_{T}\right)+\\ \left(\theta_{IP}\times \left(1-\theta_{IPS}\right)\times 3C_{T}\right) \end{array}\right]+\\ V\times \left[\theta_{IP}\times C_{I}\right]+\\ V\times \left[\theta_{PC}\times \theta_{PCS}\times \theta_{C}\times \left(C_{A}{}_{PC}+C_{FU}+C_{CT}\right)\right]+\\ V\times \left\{\begin{array}{l} \left[\theta_{IP}\times \left(1-\theta_{IPS}\right)\times \theta_{C}\times (3C_{I}+C_{FU}+C_{CT}+C_{CTI}\right]+\\ \left[\theta_{IP}\times \theta_{IPS}\times \theta_{C}\times \left(C_{A}{}_{IP}+3C_{I}+C_{FU}+C_{CT}+C_{CTI}\right)\right] \end{array}\right\} \end{array}\right\}$$ (2)

The *first expression in brackets* are the expected costs for MRSA-tests in policlinic care (first row), inpatient care given SSTI (second row), and no SSTI (third row); the *second expression in brackets* are the expected cost for precautionary measures before status is known in inpatient care; the *third expression in brackets* are the expected costs for treatment, follow-up, and contact tracing in policlinic care; the *fourth expression in brackets*, finally, are the expected costs for follow-up and contact tracing in inpatient care, given positive MRSA test but no SSTI (first row), and positive MRSA test and SSTI (second row).

The model was run using @Risk 6 (Palisade Corporation), an add-in programme to Excel. To obtain the 95 percent credibility intervals, 10 000 simulations were made.

### Expected costs of the recommendations

Top quality genetic material for pig breeding results from a continuous elaborate process. Production of genetic material from elite breeding pigs is controlled by large international companies and access to genetic material is regulated by contracts between operators at different levels in the industry and pig producers the details of which are not observable. Prices of semen for production herds are available on the companies’ homepages but there are no data on prices of boars.

At present, two companies provide the vast majority of genetic material for pig breeding in Sweden. To ensure progress both companies regularly import genetic material to their nucleus breeding herds. One of them imports live boars from Norway and the other imports semen from Denmark. However, the analysis is performed assuming that both companies import live boars from Norway.

The breeding companies are profit maximising firms relying on revenues from their produce to cover cost. A first condition for profit maximisation is that marginal revenues (*MR*) cover marginal costs (*MC*) of the operation [43, 44]. The semen produced by the imported boars may be sold directly to production herds. Alternatively, it can be used in sows in the breeding companies’ own nucleus herds, which then produce off-spring which, ultimately, produce semen and breeding pigs for the production herds. However, the latter option entails further costs and a delay of revenues. A second condition for profit maximisation is, therefore, that the semen is allocated so that the present value of *MR* (net of breeding costs) is the same regardless of use [43, 44].

Following the advice from SVA to prevent introduction of LA-MRSA entails costs to the breeding companies and to society. Some of them are straight forward while others may be less obvious. The next section illustrates how and why these costs arise.

### Profit maximisation with and without the measures suggested by SVA

**Fig 2:**
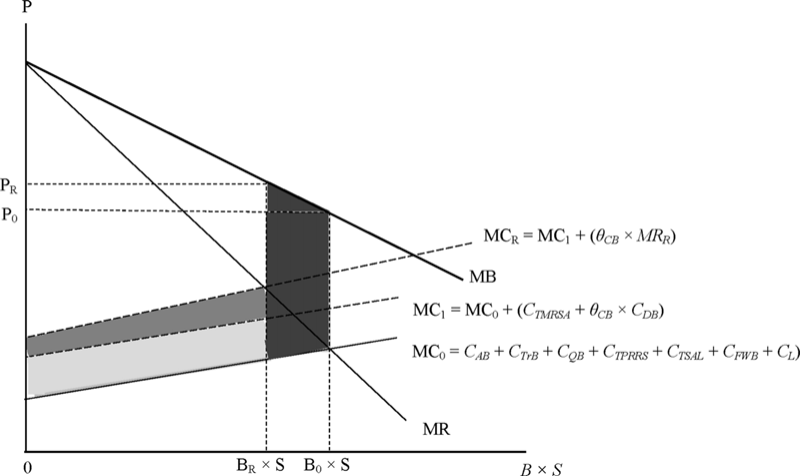
Profit maximisation with and without the preventive measures.

Fig 2 is a stylised picture of the situation facing a breeding company. A boar (*B*) produces *S* insemination doses per year. If there are no external effects, the marginal societal value of semen equals the market price (*P*). The conventional assumption of decreasing marginal values is made [43, 44]. Hence, the more semen available, the lower the price, illustrated by the line *MB*. With only two breeding companies, the market for semen is assumed to be one of monopolistic competition, or oligopoly, implying that the price is higher than the producers’ *MR* [43, 44].

In the *absence* of the measures, the marginal costs of producing semen include costs for acquiring the boar (*C*_*AB*_); transporting (*C*_*TrB*_); quarantining (*C*_*QB*_); and testing it for PRRS (*C*_*TPRRS*_) and Salmonella (*C*_*TSAL*_), mandatory in Sweden [45]; cost for accom-modation (feed and water *C*_*FWB*_, and labour costs *C*_*L*_); and for collecting the semen and administrating the sales thereof (labour costs *C*_*L*_). The conventional assumption of increasing marginal cost is made [43, 44]. Hence, the larger the production, the higher the *MC*, as illustrated by the line *MC*_*0*_. In the absence of the measures, the profit maximising quantity of semen is (B_0_×*S*) doses where *MR* equals *MC*_*0*_.

*Following the advice from SVA* will raise *MC* by the costs for the MRSA-tests (*C*_*TMRSA*_) and for the destruction of MRSA-positive boars (*C*_*DB*_, with probability *θ*_*CB*_), shifting the MC-curve to *MC*_*1*_. Also, (expected) marginal revenues fall to (1 –*θ*_*CB*_)×*MR* since only MRSA-negative boars may produce semen. Regarding the resulting revenue loss, *θ*_*CB*_×*MR*, as an additional marginal cost raises the MC-curve further to *MC*_*R*_.

Higher marginal costs imply that the breeding company will reduce semen production to (*B*_*R*_×*S*) doses, where *MR = MC*_*R*_, to maximise profits. At this level of production, the societal value of the marginal insemination dose is *P*_*R*_, i.e. higher than *P*_*0*_. Still, the reduction in quantity implies a loss to the breeding companies. Whether or not this constitutes a *societal* cost depends on if the loss of Swedish semen production can be compensated by imports of semen. To avoid underestimating costs, it is assumed that this is not the case but we return to this in the sensitivity analysis and discussion.

The costs of following the advice on prevention may, therefore, be summarised as:

1. Higher *MC* caused by the MRSA-tests, the expected culling of MRSA-positive boars and an extended quarantine period. The annual costs of these effects (light-grey area in Fig 2) are$\sum_{i=0}^{B_{R}}(MC_{1i}-MC_{0i}).$
2. Higher *MC* (reduction in *MR*) since only MRSA-negative boars produce semen. The annual cost of this effect (dark-grey area in Fig 2) is $\sum_{i=0}^{B_{R}}(MC_{Ri}-MC_{1i}).$
3. Loss of production values due to the fall in semen production due to the recommendations. The annual cost represented by the loss of these (net) production values (black area in Fig 2) is $\sum_{i=B_{R}}^{B_{0}}(MB_{i}-MC_{0i}).$

Estimation requires information on the costs of MRSA-tests, the probability that a boar is MRSA-positive, and the change in the profit maximising quantity of semen. In addition, boars of four different breeds—Hampshire, Duroc, Landrace, and Yorkshire—are involved. Prices of semen from Hampshire and Duroc are about the same but substantially lower than from Landrace and Yorkshire [46, 47]. Costs under (2) and (3) above are, therefore, calculated separately for Duroc/Hampshire and Landrace/Yorkshire.

### The probability that at least one boar tests positive, annual number of imported boars, batches, insemination doses per boar, and the prices of insemination doses

Norwegian boars are imported in batches (personal communication, breeding company). The probability that MRSA is found in a batch is assumed to equal the *herd prevalence* (*θ*_*CH*_) of MRSA. In 2012, 175 Norwegian herds were screened for MRSA. One herd was found positive [48], giving a herd prevalence of MRSA of 0.6 percent (Table 4).

**Table 4:** Probability of at least one boar being MRSA-positive, annual number of imported boars, batches, insemination doses produced per boar, and prices of insemination doses (€, 2011 prices).

Variable	Description	Value
*θ*_*CH*_	Probability that at least one boar in an batch is MRSA-positive^a^	0.006
*B*_*R*_	Total number of boars imported annually when following the recommendations^b^	400
*B*_*DHR*_	Total number of Duroc/Hampshire boars imported annually when following the recommendations^b^	222
*δ*_*DHR*_	Share of Duroc/Hampshire in total imports^b^	0.555
*B*_*LYR*_	Total number of Landrace/Yorkshire boars imported annually when following the recommendations^b^	178
*δ*_*LYR*_	Share of Landrace/Yorkshire in total imports^b^	0.445
*S*	Average number of insemination doses produced by a boar during its productive life^b^	2 340
*M*	Total number of imported batches per year^b^	22
*X*	Number of boars per import batch^b^	18
*P*_*DHR*_	Market price of semen from Duroc/Hampshire boars when breeding companies follow the recommendations (€ per insemination dose)^c^	6.20
*P*_*LYR*_	Market price of semen from Landrace/Yorkshire boars when breeding companies follow the recommendations (€ per insemination dose)^c^	30.44

^a)^Based on the herd prevalence of MRSA in Norway in 2012 [47].

^b)^Personal communication, breeding companies.

^c)^Breeding companies’ home pages [45, 46 both accessed 2014-04-23.

The company importing boars from Norway has about 35 percent of the Swedish market. It imports a total of 140 boars annually in eight batches of 18 boars each, of which 55.5 percent are Duroc/Hampshire, and 44.5 percent Landrace/Yorkshire (personal communication, breeding company). Assuming that the other company could behave similarly, a total of 400 boars (222 Duroc/Hampshire and 178 Landrace/Yorkshire) would be imported annually in 22 batches (Table 4). As both companies follow the recommendations on prevention, this corresponds to the quantity *B*_*R*_ in Fig 2.

A boar produces about 2 340 insemination doses during its productive life (personal communication, breeding companies). The price of an insemination dose given the recommendations is about € 6.20 for Duroc/Hampshire and about € 30.44 for Landrace/Yorkshire (Table 4) [46, 47]

### Costs for MRSA-tests, for the destruction of MRSA-positive batches, revenue losses from destructed boars, and revenue losses caused by reduced demand for semen

To avoid burdening the presentation with too much detail, the interested reader is referred to S1 Information for a thorough exposition of the calculations. Here, the method is presented in general terms.

MRSA-testing is performed at three separate occasions during the quarantine period. Quarantining due to testing for PRRS and Salmonella is already mandatory [45]. It is assumed that testing also for MRSA will not increase the quarantine period. Hence, following the recommendations will not affect quarantine costs.

*The costs for MRSA-tests* (*C*_*TMRSA*_) include costs for taking samples from the boars and costs for analysing the samples. In addition, two environmental samples per batch are collected and analysed. Sampling costs (*C*_*sampl*_) are about € 14.11 per sample and costs for analysis (*C*_*anal*_) about € 78.60 per analysis, (Table 5; personal communication, SVA, SJV and SvDHV). Up to five individual samples may be pooled and analysed for the same cost as one (personal communication, SVA). This makes it impractical to calculate marginal costs for MRSA-tests and incremental (costs per batch) are used instead.

**Table 5:** Cost for MRSA-test, destruction of MRSA-positive boars (€, 2011 prices) and price elasticity.

Variable	Description	Value
*C*_*sampl*_.	Costs for taking samples for MRSA-tests (per sample)^a^	14.11
*C*_*anal*_.	Costs for analysis of MRSA samples^b^	78.60
*C*_*DB1*_	Costs for destruction of the first MRSA-positive boar in a batch^c^	54.86
*C*_*DB2*_	Costs for destruction of additional MRSA-positive boars in a batch (per boar) ^c^	41.14
*ε*_*P*_	Price elasticity of the demand for pork^d^	- 0.534

^a)^Personal communication SJV and SvDHV.

^b)^ Personal communication SVA.

^c)^Personal communication Svensk Lantbrukstjänst.

^d)^Breeding companies’ homepages (http://avelspoolen.se and http://www.qgenetics.se) both accessed 2014-04-23.

*The costs for destruction of MRSA-positive boars* are € 54.86 for the first animal (*C*_*DB1*_) and then € 41.14 (*C*_*DB2*_) for each additional boar (Table 5; personal communication, Svensk Lantbrukstjänst). Thus, again incremental costs are used instead of marginal costs.

Estimation of *the loss of revenues caused by culling* all boars in a batch where at least one of them is MRSA-positive requires information of *MR* at the optimal quantity of semen. As we don’t know the shape of the *MR*-function, this is unknown. On the other hand, *MR* cannot exceed the price of semen. Hence, though this will overstate the costs represented by the dark-grey area in Fig 2, semen prices are used to approximate *MR* from the respective breeds at the optimal number of doses,

The *revenue loss caused by the reduction in the demand for semen* resulting from the increase in production costs (represented by the black area in Fig 2) is approximated as follows:

*First*, assuming that semen production is infinitely sensitive to changes in costs (i.e. if *MC* should increase, production would fall to zero unless producers are *fully* compensated [43, 44]), the price change, for each breed *j* (Δ*P*_*j*_), needed to compensate producers is calculated. *Second*, to calculate how much this price increase will reduce the demand for semen requires information on how sensitive it is to price changes. To our knowledge this has not been estimated. It is therefore approximated by the price elasticity of the demand for pork (*ε*_*P*,_ as demand for semen is derived from the demand for pork) In Sweden, *ε*_*P*_ has been estimated to—0.534 [49] (Table 5), implying that a price increase of one percent will reduce the demand for pork by 0.534 percent. *Third*, as information on *MC*_*0*_ is the property of the companies the costs represented by the black area in Fig 2 between the *MB* and *MC*_*0*_-curves from (*B*_*R*_×*S*) to (*B*_0_×*S*) cannot be calculated precisely. Instead, the whole area under the *MB*-curve from (*B*_*R*_×*S*) to (*B*_0_×*S*) is calculated. As this also includes the area *under* the *MC*_*0*_-curve, i.e. costs that would have been incurred even in the absence of the measures, it may overstate the net loss. On the other hand, if the demand for semen is more sensitive to price changes than the demand for pork, the reduction in demand would be larger and the loss of societal welfare due to the reduction in semen production understated.

### Total expected costs of the measures suggested by SVA

The expression for the total expected societal costs of the preventive measures *E*(*SC*_*Rec*_), i.e. the sum of the three shaded areas in Fig 2, is:

$$E\left(SC_{\mathrm{Pr}ev}\right)=\left\{\begin{array}{l} M\times \left\{3\times \left[X\times C_{sampl}+\left(\frac{X}{5}\times C_{anal}\right)\right]+2\times \left(C_{sampl}+C_{anal}\right)\right\}+\\ M\times \theta_{CH}\times \left\{C_{DB1}+\left(X-1\right)\times C_{DB2}\right\}+\\ \left[\theta_{CH}B_{R}S\left(\delta_{DHR}P_{DHR}+\delta_{LYR}P_{LYR}\right)\right]+\\ \left[\left(P_{DH0}\Delta \left(B_{DH}S\right)+\Delta P_{DH}\frac{\Delta \left(B_{DH}S\right)}{2}\right)+\left(P_{LY0}\Delta \left(B_{LY}S\right)+\Delta P_{LY}\frac{\Delta \left(B_{LY}S\right)}{2}\right)\right] \end{array}\right\}$$ (4)

where the first row are the costs for MRSA-test, and the second are the expected costs for destruction of MRSA-positive boars which together make up the light-grey area in Fig 2., The third row is the expected revenue loss from destructed MRSA-positive boars, i.e. the dark-grey area in Fig 2.,The fourth row is the revenue loss caused by the reduction in the demand for semen (from, respectively, Duroc/Hampshire and Landrace/Yorkshire), i.e. the black area in Fig 2. For details, see S1 Information.

## Baseline Results and Sensitivity Analysis

### Baseline results

Table 6 shows the estimated annual number of visits (rounded to integers) of patients belonging to the risk group in each part of the event tree in Fig 1 given Danish and Dutch human prevalence, estimated to be 8 percent and 15 percent respectively.

**Table 6:** Expected annual number of visits by patients in the risk group in each part of the event tree given Danish and Dutch prevalence (rounded to integers).

*Prevalence independent*		
Total number of visits (*V*)	9303	
number of visits treated policlinically: (*V*×*θ*_*PC*_)	8685	
number of visits referred to inpatient care: (*V*×*θ*_*PC*_)	618	
number of SSTI’s treated policlinically: (*V*×*θ*_*PC*_× *θ*_*PCS*_)	132	
number of inpatient visits with no SSTI: [*V*×*θ*_*IP*_×(1*-θ*_*IPS*_)]	615	
number of inpatient visits with SSTI: (*V*×*θ*_*IP*_×*θ*_*IPS*_)	3	
*Prevalence dependent*	*Danish prevalence*	*Dutch prevalence*
number of LA-MRSA-positive visits, policlinics:(*V*×*θ*_*PC*_×*θ*_*PCS*_×*θ*_*C*_)	11	20
number of LA-MRSA-positive visits, inpatient care, no SSTI: [*V*×*θ*_*IP*_×(1*-θ*_*IPS*_)×*θ*_*C*_]	49	95
number of LA-MRSA-positive visits, inpatient care, SSTI:(*V*×*θ*_*IP*_×*θ*_*IPS*_×*θ*_*C*_)	0.2	0.4

Using the quantities in Table 6, and assuming Danish human prevalence, the total expected annual cost savings from the preventive measures in steady state, are estimated to about € 870 700 (95% credibility interval: € 604 233.8 –€ 1 170 255.7) while, assuming Dutch human prevalence, they are estimated to about € 1 233 500 (95% credibility interval: € 953 262.9 –€ 1 544 133.6) in 2011 prices. Table 7 presents these benefits according to where they arise. It may be noted that the two largest components are the costs caused by precautionary measures before diagnosis in inpatient care (which are independent of prevalence), and costs for contact tracing in inpatient care for patients without SSTI.

**Table 7:** Expected benefits (95 percent credibility intervals) when preventing LA-MRSA from being introduced into the Swedish pig population assuming human prevalence as in, respectively, Denmark and the Netherlands (€, 2011 prices).

	Expected benefits			
	Human prevalence as in Denmark		Human prevalence as in the Netherlands	
Diagnostic tests, PC, SSTI^a^	5693.7	(4929.2–6675.1)	5693.7	(4929.2–6675.1)
Diagnostic tests, IP, No SSTI^a^	81563.8	(72194.1–93726.0)	81563.8	(72194.1–93726.0)
Diagnostic tests, IP, SSTI^a^	480.6	(399.9–572.3)	480.6	(399.9–572.39)
Precautionary measures before diagnosis, IP^a^	393804.8	(348593.4–452583.4)	393804.8	(348593.4–452583.4)
*Sub-total diagnostics and precaution before diagnosis*	*481543*.*1*	*(426256*.*3–553223*.*8)*	*481543*.*1*	*(426256*.*3–553223*.*8)*
Treatment, PC, SSTI	No excess costs compared to sensitive infections		No excess costs compared to sensitive infections	
Follow-up, PC, SSTI	15635.9	(5397.1–26446.8)	30183.1	(19786.4–41528.5)
Contac tracing, PC, SSTI	15000.9	(4911.5–26952.8)	28937.3	(17517.2–42553.2)
*Sub-total treatment*, *follow-up and contact tracing*, *PC*	*30636*.*4*	*(10498*.*2–53001*.*4)*	*59120*.*4*	*(38139*.*0–82553*.*2)*
Precautionary measures, IP, No SSTI	94 228.9	(32820.2–158808.5)	181890.4	(120348.3–248194.9)
Follow-up, IP, No SSTI	74365.3	(25743.8–125831.4)	143539.7	(9 978.7–195874.9)
Contact tracing, IP, No SSTI	188494.0	(64401.0–322666.6)	363732.8	(237898.3–502519.7)
*Sub-total*, *IP*, *No SSTI*	*357088*.*2*	*(122800*.*2–604902*.*6)*	*689152*.*8*	*(453625*.*6–947 717*.*8)*
Precautionary measures, IP, SSTI	418.1	(142.2–712.4)	807.0	(517.4–1119.6)
Treatment, IP, SSTI	16.5	(5.6–28.1)	31.8	(20.6–44.1)
Follow-up, IP, SSTI	329.9	(112.2–566.3)	636.9	(408.3–883.6)
Contact tracing, IP, SSTI	836.3	(283.7–1433.7)	1613.9	(1039.6–2267.9)
*Sub-total*, *IP*, *SSTI*	*1600*.*8*	*(543*.*1–2727*.*5)*	*3089*.*5*	*(1991*.*8–4311*.*5)*
**Total**	**870727.0**	**(**604233.8–1170255.7)	**1233510.9**	**(**953262.9–1544133.6)

^a^ Prevalence independent

The total expected annual societal costs of the recommendations are found to be € 211 128.6. In Table 8, these costs are presented according to where they arise. As can be seen, the largest contribution is from the loss of revenues from destructed LA-MRSA positive boars.

**Table 8:** Expected annual costs of following the recommendations (€, 2011 prices).

Cost-category	Value
MRSA-tests:	41862.19
Destruction of MRSA-positive boars:	100.55
Revenue loss caused by destruction of boars:	95397.87
Loss of production values caused by reduced demand for semen due to cost increase:	73868.56
**Total annual costs:**	**211128.6**

Thus, given the assumptions in the study, the results indicate that the preventive measures suggested by SVA would increase societal welfare.

### Sensitivity analysis

As a the herd prevalence of 0.6 percent probably is lower than in most EU-countries, and since the herd prevalence affects all costs except those for the MRSA-tests, it is of interest to investigate how high *θ*_*CH*_ could be without causing the societal costs to exceed the societal benefits of the recommendations.

This “break-even” level of prevalence is found by setting the minimum values of societal benefits in Table 7 equal to the expression for the societal costs in Eq (4) and solve for *θ*_*CH*_. Accordingly, the break-even rates of the herd prevalence (all other things equal) are as in Table 9 below:

**Table 9:** Break-even rates of herd prevalence of LA-MRSA in the exporting country.

Human prevalence of CC398 from	Lower limit of benefits from Table 7 (€)	Break-even *θ*_*CH*_
Denmark	610861.6	0.0221
The Netherlands	1001024.4	0.0362

That is, if steady state prevalence of LA-MRSA in the Swedish human risk group reaches the Danish (Dutch) level, the benefits of the measures are large enough to cover their costs if the herd prevalence in the country from which they are imported does not exceed 2.21 (3.62) percent.

As there also is uncertainty regarding how sensitive the demand for semen is with respect to price changes it is of interest to investigate how large the price elasticity could be without causing the societal cost to exceed the societal benefits of the measures. Thus, setting the minimum values of societal benefits in Table 7 equal to the expression for the societal costs in Eq (4) and solving for price sensitivity *ε*, the break-even levels are (Table 10):

**Table 10:** Break-even level of price elasticity of demand for semen.

Human prevalence of CC398 from	Lower limit of benefits from Table 7 (€)	Break-even *ε*
Denmark	610861.6	– 3.43
The Netherlands	1001024.4	– 5.99

Accordingly, other things equal, the (absolute value of) price sensitivity of the demand for semen could be as high as 3.43 (5.99), given Danish (Dutch) human prevalence, without the costs of the preventive measures exceeding their benefits.

In the baseline, the risk group includes persons in close contact with pigs only. According to results from Denmark [16] about 21 percent of detected LA-MRSA had no known direct contact with live pigs. If this should apply to Sweden, there would be about 16 (31) additional LA-MRSA positive visits annually given Danish (Dutch) human prevalence in the risk group. Assuming that they are distributed between policlinic and inpatient care like LA-MRSA positive visits in the risk group (see Table 6), and using the costs in Table 3, expected cost savings in human health care would increase to about € 927 500 (€ 1 344 200) other things equal.

An anonymous reviewer pointed out that there are more recent studies of LA-MRSA among pig farmers in some EU-countries, including Denmark and the Netherlands [50, 51] arriving at higher estimates of prevalence. Thus, if prevalence among Swedish pig farmers would be as high as that found among Dutch pig farmers in these two studies (75 and 38 percent respectively), the expected cost savings in Swedish human health care would increase to € 2 171 910 or € 1 431 369 other things equal.

On the cost side, if the loss of domestic semen production could be replaced by imports of similar quality, it may be argued that it would not represent a societal loss in the long run. This is because Swedish production herds still would be supplied with genetic material (albeit by foreign breeding companies) implying that there would be no loss in pork production, and because the resources in Swedish breeding companies made redundant by the production cuts would be expected to find employment in other sectors. In that case, the costs of the preventive measures in Table 8 would be reduced by € 73 868.56 to € 121 027.4.

## Discussion

The spread of LA-MRSA is global and such bacteria are common among pigs and other farm animals in several countries [1]. However, to our knowledge, few actions have been taken to control the spread of LA-MRSA in pigs. The reasons for this can only be speculated on, but most likely the widespread occurrence of LA-MRSA when first discovered in 2004 is an important factor. In Sweden, LA-MRSA in pigs is rare [8] and interventions to keep prevalence low might still be feasible. An example of such interventions is the measures suggested by SVA analysed in the present study.

Our baseline results indicate that the benefits of the suggested measures (€ 870 727 –€ 1 233 510) exceed their costs (€ 211 128), giving a net societal benefit of between € 659 599 and € 1 022 382 depending on the expected human prevalence of LA-MRSA if it became endemic. Thus, given the assumptions made in the study, the measures generate a net societal benefit and, therefore, appear to be feasible for preventing the introduction and spread of LA-MRSA among pigs in Sweden.

When estimating the baseline results, our strategy has been to avoid overestimating the benefits and avoid underestimating the costs of the measures. Hence, on the benefit side, only persons expected to frequently be in contact with live pigs are considered to be at risk of being colonised or infected. This results in a risk group of about 6 000 persons only. It is also assumed that introduction of LA-MRSA among Swedish pigs would not result in a larger number of *S*. *aureus* infections in the risk group, implying that only the excess costs caused by bacteria being resistant are considered. When estimating these excess costs, it is assumed that the antibiotics used for treatment would be efficient (that is, potential costs for extended treatment and hospital stay are not considered). Furthermore, costs caused by additional cases revealed during contact tracing are not included. For the same reason, the value of avoiding pain and anxiety, of avoiding restrictions on the activities of infected persons, or of avoiding premature deaths caused by LA-MRSA in the risk group are also not included.

On the cost side, the expected loss of marginal revenues caused by the fact that only non-positive boars may produce semen, are overestimated by the use of the market price of semen as an approximation for the unobservable marginal revenues. In addition, the expected loss of marginal revenues, and the expected costs for destruction of boars due to positive findings of LA-MRSA may also be overestimated by using the average herd prevalence in the exporting country as a proxy for the probability of at least one boar testing positive in an import batch originating from a herd in the top of the breeding pyramid.

Our sensitivity analysis shows that the expected costs of having LA-MRSA in the pig population could be substantially higher if colonisation spread outside the risk group or if prevalence were higher than in our baseline assumptions. It also shows that the costs of the suggested measures are smaller than in the baseline if the loss of Swedish semen production could be substituted by imports of similar quality. However, direct import of semen by Swedish production herds is almost non-existent, suggesting that the loss of semen production in domestic breeding companies should be regarded a societal cost.

Nevertheless, it might be tempting to regard the baseline results as a conservative estimate of the societal benefit of the recommendations. Still, they rest on a number of assumptions that need to be discussed.

The most crucial of these is, perhaps, that the suggested measures actually would *prevent* the introduction of LA-MRSA among Swedish pigs. Although trade and transfer of live animals is considered the most important risk factor for spread of LA-MRSA [52–54], there might be other routes for introduction such as persons working at or visiting farms [52, 53, 55], and, possibly, by air [56]. LA-MRSA has also been found in horses in Sweden and transfer from horses to pigs through persons in contact with both species is a possible scenario. However, screening of horses has shown that MRSA is rare in healthy horses in Sweden [8]. Moreover, horses with confirmed MRSA are to be isolated during the period of clinical infection according to Swedish legislation. Accordingly, although there is a risk that MRSA can be introduced to a herd through other routes (particularly in countries where LA-MRSA is wide-spread) we assess this risk to be substantially lower than the risk for introduction through trade of colonised pigs. One argument for this is that none of the studies above have been able to quantify it. Nevertheless, to ensure that an MRSA free pig population is upheld, it would be necessary to complement testing of imported breeding pigs with regular monitoring of pig herds and biosecurity measures on herd level. Costs for monitoring or potential intervention are not included in the present study as they will depend on type and frequency of measures considered which, in turn, depends on how high the risk of introduction through other routes is. Accordingly, information on this risk is central to whether or not LA-MRSA free countries should apply preventive measures and how these should be designed.

Second, the assumed probability of a boar testing positive for LA-MRSA is uncertain. Our sensitivity analysis shows that, should it be larger than 2.31 (3.71) percent, the expected annual costs of the recommendations would exceed the expected annual gains from a smaller number of MRSA cases in human health care. According to the study by EFSA [22], the average prevalence of LA-MRSA in breeding herds in the EU in 2008 was 13 percent, varying between member states from 0 to 46 percent, and 12 member states did not detect LA-MRSA. Although the prevalence of MRSA in most member states has increased since 2008 there are probably herds that are free of LA-MRSA. Thus, given that those herds could be identified, the risk of positive test results could be kept below the critical level. However, information of which herds that are free of LA-MRSA is not readily available.

Third, the prevalence in the human risk group is assumed to be between 8 and 15 percent based on the Danish and Dutch data. However, between 2009 and 2013 the number of reported human cases of LA-MRSA in Denmark has increased from 232 to 643 cases [57]. This suggests an increased prevalence and accordingly the societal benefits using the Danish prevalence in this study could have been underestimated.

Our assumption of LA-MRSA not being very pathogenic to humans should also be considered. It is based on findings in the literature that human transmission of LA-MRSA and incidence of clinical infections in humans is lower than for other types of MRSA [58–61]. The reason may be bacterial factors such as reduced virulence of the strains and host factors such as a risk group consisting of mainly healthy people [2, 58]. Nevertheless, the potential to cause disease in humans is substantial [57, 59] and there are several reports of clinical disease in humans [62–64]. Also, in the present study we did not include the costs that would arise in human health care if the introduction of LA-MRSA was to result in a larger number of *S*.*aureus* infections in the risk group or in the population at large.

Having a pool of resistance genes in the pig population constitutes another risk that is hard to estimate the importance of and which costs, consequently, have not been estimated. There is a potential for transfer of resistance genes to *S*. *aureus* strains that are more pathogenic for humans. Moreover, there are concerns of a shift in virulence and a re-adaptation of LA-MRSA to humans which would generate a huge reservoir of human adapted MRSA in livestock [2]. The probability of these events could not be quantified, the consequences should they occur, however, would be serious.

## Conclusions

Having LA-MRSA in the pig population causes significant societal costs. Given the assumptions in the study, the results indicate that measures to prevent LA-MRSA from becoming endemic increase societal welfare. However, given the significant gaps in knowledge, it is suggested that a complete cost benefit analysis should be done in the future as more data become available.

## Supporting Information

S1 Information **Table A.** Data used for estimation of probabilities. **Table B.** Data used for estimation of LA-MRSA prevalence in the Dutch risk group (*θ*_*CNL*_).
(DOCX)
