# Supplementary material for: Preventing Introduction of Livestock Associated MRSA in a Pig Population – Benefits, Costs, and Knowledge Gaps from the Swedish Perspective
Source: PLoS One. 2015 Apr 29;10(4):e0122875. doi: 10.1371/journal.pone.0122875 (PMC4414519; doi:10.1371/journal.pone.0122875)
Supplement: S1 Information — Table A. Data used for estimation of probabilities. Table B. Data used for estimation of LA-MRSA prevalence in the Dutch risk group (θ CNL). (DOCX) [file pone.0122875.s001.docx]

**Supporting information**

**Data**

Table A shows the data used to estimate the probabilities *θ_PC_*, *θ_IP_*, *θ_IPS_*, and (1- *θ_IPS_*).

**Table A. Data used for estimation of probabilities.**

| **Year** | **PC-visits/ 100 000^a^** | **IP-visits/ 100 000^b^** | **PC-visits SSTI/ 100 000^c^** | **IP-visits SSTI/ 100 000^b^** |
| --- | --- | --- | --- | --- |
| 2011 | 156500 | 10309 |  | 50 |
| 2010 | 153200 | 10494 |  | 47 |
| 2009 | 149700 | 10387 |  | 46 |
| 2008 | 147500 | 10252 |  | 49 |
| 2007 | 143900 | 10148 |  | 45 |
| 2006 | 142800 | 10150 |  | 43 |
| 2005 | 140600 | 10025 | 1880 | 42 |
| 2004 | 131900 | 10018 | 2167 | 43 |
| 2003 | 131100 | 9988 | 2089 | 42 |
| 2002 | 137500 | 10020 |  | 44 |
| 2001 | 137200 | 10157 |  | 42 |

^a)^Swedish local communities and county councils. ^b)^Swedish Board of Health and Welfare. ^c)^André et al. (2008).

Table B shows the data used for estimating LA-MRSA prevalence in the Dutch risk group.

**Table B. Data used for estimation of LA-MRSA prevalence in the Dutch risk group $(\theta_{CNL})$.**

| **Study** | **Persons in study** | **No. of LA-MRSA-positive in study** |
| --- | --- | --- |
| Voss et al 2005 pig farmers + employees | 26 | 6 |
| v. den Broek et al 2009 pig farmers | 98 | 28 |
| van Cleef et al 2010a pig farmers | 49 | 13 |
| v. den Broek et al 2009 family members | 134 | 5 |
| van Cleef et al 2010b slaughter workers | 93 | 14 |
| Gilbert et al 2012 slaughter workers | 36 | 4 |
| Gilbert et al 2012 slaughter workers | 34 | 3 |
| Wulf et al 2007 veterinarians | 26 | 6 |

**Prevalence in the Danish risk group**

Data for Denmark are scarce and only contain information on the annual number of *reported* human cases for a few years (personal communica­tion, Department of Food and Resource Economics (FOI), Denmark). Hence, $\theta_{CDK}$, is estimated as:

$\theta_{CDK}=\frac{N_{RDK}/\lambda}{N_{DK}}$ (1)

where *N_DK_* = no. of persons in the Danish risk group, *N_RDK_* = no. of reported MRSA-positive persons in the Danish risk group, and *λ* = the proportion of the Danish risk group that is tested.

*λ* is assumed to be the same as in Sweden, calculated by dividing the expected number of persons tested annually in Sweden (342) by the number of persons in the Swedish risk group (6 080). The expected number of *persons tested* annually is obtained by dividing the expected annual number of visits where an MRSA-test is done $[V\times(\theta_{PC}\times\theta_{PCS}+\theta_{IP})=749]$ by the expected annual number of visits per person for those who make at least one visit (2.19 per year based on data from SKL for the period 2007-2011). This gives a $\lambda$ of $(342/6080=0.056)$. Combining this with information on *N_RDK_* and *N_DK_*, respectively, 149 and 22 740 persons in 2011 (personal communication FOI, Denmark), gives the estimate of $\theta_{CDK}$ reported in Table 2 in the main text.

**Calculating the costs of the recommendations**

**The probability that at least one boar is MRSA positive**

Norwegian boars are imported in batches (personal communication, breeding company). The probability that MRSA is found in a batch is assumed to equal the *herd prevalence* $(\theta_{CH})$of MRSA. In 2012, 175 Norwegian herds were screened for MRSA. One herd was found positive, giving a herd prevalence of MRSA of 0.6 percent.

It is recognized that the herds exporting boars are at the top of the breeding pyramid and more secluded and less likely to be MRSA-positive than the average herd. Moreover, even if the *herd* is MRSA-positive, a batch does not necessarily include a colonized animal. Thus, the risk of losing a batch is probably lower than 0.6 percent but, as there is no information on the risk of a boar being MRSA-positive$(\theta_{CB})$, the herd prevalence $\theta_{CH}=0.006$ listed in Table 4 of the main text is used to calculate the expected costs.

**Costs for MRSA tests and for the destruction of MRSA positive boars**

Costs for MRSA-tests (*C_TMRSA_*) include costs for taking samples from the boars and costs for analysing the samples. In addition, two environmental samples per batch are collected and analysed. As up to five individual samples may be pooled and analysed for the same cost as one, it is impractical to cal­culate marginal costs and incremental costs (costs per batch) are used in­stead. The MRSA-tests’ contribution to the annual costs is:

$\sum_{i=0}^{B_{R}} C_{TMRSA}=M\times\left\{3\times\left[X\times C_{sampl}+\left(\frac{X}{5}\times C_{anal}\right)\right]+2\times(C_{sampl}+C_{anal})\right\}$ (2)

where *M* is the total number of batches imported annually, and *X* is the number of boars per batch. Since *X* = 18 in the present case, the quota (*X*/5) is rounded to 4 to account for the indivisibility caused by the pooling of samples for analysis.

Costs for the destruction of MRSA-positive boars (*C_DB_*) are higher for the first animal (*C_DB1_*) than for the remaining boars in a batch (*C_DB2_* per boar). Thus, again incremental costs are used instead of mar­ginal costs, implying that the contribution to the annual cost from the destruction of MRSA-positive boars is:

$\sum_{i=0}^{B_{R}} C_{DB}=M\times\theta_{CH}\times\left\{C_{DB1}+\left(X-1\right)\times C_{DB2}\right\}$ (3)

The total annual costs for MRSA-test and destruction of positive batches (represented by the light-grey area in Fig. 2 in the main text) are the sum of eq. (S1) and (S2).

**Loss of revenues due to the destruction of MRSA positive boars**

Estimation of the loss of revenues caused by following the advice that all boars in a batch where at least one of them is MRSA-positive should be culled (represented by the dark-grey area in Fig. 2 in the main text) requires infor­mation of *MR* at the optimal quantity of semen. The profit maxi­mising number of boars, the prices of semen *given* the preventive measures, and the quantity of semen produced by a boar of either breed is known. However, as we don’t know the shape of the demand function from which the *MR*-function is derived, *MR* at the quantity $(B_{R}\times S)$is un­known. On the other hand, *MR* cannot exceed the price of se­men. Hence, *P_DHR_* and *P_LYR_* are used as approximations of *MR* from the respective breeds at the optimal number of doses, though it will overstate the costs represented by the dark-grey area in Fig. 2, they are calculated as:

$\sum_{i=0}^{B_{R}} MR_{i}=\theta_{CH}\times B_{R}\times S\times\left\{\left[\delta_{DHR}\times P_{DHR}\right]+\left[\delta_{LYR}\times P_{LYR}\right]\right\}$ (4)

where $\delta_{DHR}$ is the share of Duroc/Hampshire, and $\delta_{LYR}$ the share of Landrace/Yorkshire boars.

**Loss of semen caused by higher production cost**

Estimation of the costs caused by the loss of semen resulting from the increase in pro­duction costs (represented by the black area in Fig. 2 in the main text) is more complicated because it requires information on the shape of the *MB* and *MC*-curves. As this is lacking, the loss is approximated using a three step procedure:

*First*, it is assumed that semen production is infinitely sensitive to changes in costs (i.e. if *MC* should increase, production would fall to zero unless producers are *fully* compen­sated. The price change, for each breed *j,* needed to compensate producers is $\Delta P_{j}=MC_{jR}-MC_{j0}.$ To calculate $\Delta P_{j},$ the costs in eq. (S1), (S2) and (S3) are summed and multiplied by each breed’s share of production and, then, divided by the amount of semen produced by that breed. This gives:

$\begin{matrix} \Delta P_{DH}=\frac{\delta_{DHR}\left\{\left[(B_{R}C_{TMRSAl})+(\theta_{CH}B_{R}C_{DB})\right]+\left[\theta_{CH}B_{R}S(\delta_{DHR}P_{DHR}+\delta_{LYR}P_{LYR})\right]\right\}}{\delta_{DHR}\times B_{R}\times S} \\ \Delta P_{LY}=\frac{\delta_{LYR}\left\{\left\{\left[(B_{R}C_{TMRSAl})+(\theta_{CH}B_{R}C_{DB})\right]+\left[\theta_{CH}B_{R}S(\delta_{DHR}P_{DHR}+\delta_{LYR}P_{LYR})\right]\right\}\right\}}{\delta_{LYR}\times B_{R}\times S} \end{matrix}$ (5)

*Second*, to calculate how much this price increase will reduce the demand for semen, one needs information on how sensitive the demand for semen is to price changes. To our knowledge this has not been estimated. However, as demand for semen is derived from the demand for pork, it is approximated by the price elasticity of the demand for pork:

$\varepsilon_{P}=\frac{dQ/Q}{dP/P}$ (6)

where *dQ*/*Q* is the relative change in the demand for, and *dP/P* is the relative change in the price of, pork.

Thus, the fall in the demand for semen from each breed caused by the price increase is cal­culated by multiplying the relative increase in the price of semen for that breed with the price elasticity of the demand for pork and the breed’s share total boar import:

$\begin{matrix} \Delta(B_{DH}\times S)=\frac{\Delta P_{DH}}{P_{DHR}}\times\varepsilon_{P}\times\left(\delta_{DHR}\times B_{R}\times S\right) \\ \Delta(B_{LY}\times S)=\frac{\Delta P_{LY}}{P_{LYR}}\times\varepsilon_{P}\times\left(\delta_{LYR}\times B_{R}\times S\right) \end{matrix}$ (7)

*Third*, the costs represented by the black area in Fig. 2 are the *net* loss of societal welfare due to the loss of production caused by the price increase (the area *between* the *MB* and *MC_0_*-curves). This cannot be calculated as information on *MC_0_* is the property of the companies. Instead, the area under the *MB*-curve from $(B_{0}\times S)$to $(B_{R}\times S)$is calculated:

$\left\{\begin{matrix} \left[P_{DH0}\times\Delta(B_{DH}\times S)+\Delta P_{DH}\times\frac{\Delta(B_{DH}\times S)}{2}\right]+ \\ \left[P_{LY0}\times\Delta(B_{LY}\times S)+\Delta P_{LY}\times\frac{\Delta(B_{LY}\times S)}{2}\right] \end{matrix}\right\}$ (8)

As this includes the area *under* the *MC_0_*-curve from$(B_{0}\times S)$to $(B_{R}\times S)$ in Fig. 2, i.e. costs that would have been incurred also in the absence of the measures, it may over­state the net loss. On the other hand, if the demand for semen is more sensitive to price changes than the demand for pork, the reduction in demand would be larger and the loss of societal welfare due to the reduction in semen production understated.
